# Chorismate mutase peptide antibody enables specific detection of *Acanthamoeba*

**DOI:** 10.1371/journal.pone.0250342

**Published:** 2021-04-23

**Authors:** Hae-Ahm Lee, Ki-Back Chu, Min-Jeong Kim, Fu-Shi Quan, Hyun-Hee Kong, Eun-Kyung Moon

**Affiliations:** 1 Medical Research Center for Bioreaction to Reactive Oxygen Species and Biomedical Science Institute, School of Medicine, Graduate School, Kyung Hee University, Seoul, Korea; 2 Department of Biomedical Science, Graduate School, Kyung Hee University, Seoul, Korea; 3 Department of Medical Zoology, Kyung Hee University School of Medicine, Seoul, Korea; 4 Department of Parasitology, Dong-A University College of Medicine, Busan, Korea; Instituto Butantan, BRAZIL

## Abstract

Accurate and rapid diagnosis of *Acanthamoeba* keratitis (AK) is difficult. Although the diagnostic procedure for AK has improved, further development and effective diagnostic tool utilization for AK need to continue. Chorismate mutase is a key regulatory enzyme involved in the shikimate pathway, a metabolic pathway absent in mammals but central for amino acid biosynthesis in bacteria, fungi, algae, and plants. In this study, we describe the identification and production of a polyclonal peptide antibody targeting chorismate mutase secreted by *A*. *castellanii*, which could be used for AK diagnosis. Western blot was performed using the protein lysates and conditioned media of the human corneal epithelial (HCE) cells, non-pathogenic *Acanthamoeba*, pathogenic *Acanthamoeba*, clinical isolate of *Acanthamoeba* spp., and other causes of keratitis such as *Fusarium solani*, *Pseudomonas aeruginosa*, and *Staphylococcus aureus*. Polyclonal antibodies raised against *A*. *castellanii* chorismate mutase specifically interacted with lysates of *Acanthamoeba* origin and their culture media, while such interactions were not observed from other samples. *Acanthamoeba*-specificity of chorismate mutase was also confirmed using immunocytochemistry after co-culturing *Acanthamoeba* with HCE cells. Specific binding of the chorismate mutase antibody to *Acanthamoeba* was observed, which were absent in the case of HCE cells. These results indicate that the chorismate mutase antibody of *Acanthamoeba* may serve as a method for rapid and differential *Acanthamoeba* identification.

## Introduction

*Acanthamoeba* keratitis (AK) is a painful and sight-threatening infection caused by several free-living amoebae belonging to the genus *Acanthamoeba* [1]. Over the past few decades, the incidence rates of AK have been increasing and several risk factors associated with AK have been delineated, which includes contact lens usage and corneal trauma [2]. Although early detection and diagnosis of AK could lead to successful treatment, accurately diagnosing AK remains difficult and this has frequently resulted in ocular *Acanthamoeba* infection being misdiagnosed as an infection of viral, bacteria, or fungal origin [1–3]. Inaccurate or delayed diagnosis can lead to AK treatment failure. Current diagnostic methods for AK primarily rely on microbiological culture and microscopic identification, while histochemical staining and PCR-based diagnosis are also available [4–7]. Nevertheless, these methods require corneal scrapings that inflict immense pain to the patients during the sample acquisition process and as such, a non-invasive diagnostic method encompassing a high degree of sensitivity and *Acanthamoeba* specificity is desired.

The potential of highly specific antibody-based *Acanthamoeba* detection methods has previously been reported [8, 9]. Several studies involving specific antibodies against *Acanthamoeba* spp. for AK diagnosis have reported interesting results. *Acanthamoeba*-specific lacrimal IgA and serum IgG were detected from both AK patients and healthy subjects, with lesser quantities of lacrimal IgA being reported from the former of the two [10]. Four antibody clones that specifically bind to *Acanthamoeba* spp. were isolated from a bacteriophage display library. Flow cytometry and immunofluorescence data revealed that the clone HPPG6 demonstrated specific binding to *A*. *palestinensis* [8]. Monoclonal antibodies targeting *A*. *castellanii* have been produced and their interactions with *Acanthamoeba* trophozoite or cyst were confirmed by flow cytometric analysis [9]. A monoclonal antibody raised against a mannose-binding protein of *A*. *culbertsoni* exhibited cross-reactivity with other *Acanthamoeba* spp. [11]. Although these earlier findings highlighted the importance of antibodies for AK diagnosis, most of the aforementioned studies did not investigate the interspecies interaction that may occur between *Acanthamoeba*-specific antibodies and other causative agents of keratitis. Recently, an immunochromatography-based assay kit using fluorescent silica nanoparticles was developed for rapid AK diagnosis [12]. Ease of use and rapid *Acanthamoeba* detection are the major advantages of this method, but the kit suffers from low sensitivity for cyst detection. Furthermore, the *Acanthamoeba*-specificity of this detection method requires elucidation. To this extent, developing an AK diagnostic method that encompasses the favorable features of these earlier works whilst demonstrating *Acanthamoeba*-specificity would be promising.

In this study, we sought to develop a rapid and simple diagnostic method for AK using *Acanthamoeba*-specific antibodies. We isolated the chorismate mutase protein from the secretory proteins of pathogenic *A*. *castellanii*. Chorismate mutase is an enzyme that catalyzes the chemical reaction of chorismate to prephenate in the shikimate pathway, which are subsequently used for producing the amino acids phenylalanine and tyrosine [13, 14]. This pathway, originally discovered in plants, was later reported to be present in various other organisms such as the bacterium *Mycobacterium tuberculosis*, fungi, and parasites [14–18]. Since the chorismate mutase is absent in humans and other mammals, this enzyme could serve as a potential target for antimicrobial agent development [19, 20]. The present study aimed to discover a chorismate mutase-specific antibody of *A*. *castellanii* origin, which can be used for *Acanthamoeba* identification. The results of this study were supported by western blot analysis and immunocytochemistry.

## Materials and methods

### Cell culture

*Acanthamoeba*, human corneal epithelial (HCE) cells, *Fusarium solani*, *Pseudomonas aeruginosa*, and *Staphylococcus aureus* were prepared as previously described [21]. Briefly, both of the non-pathogenic (ATCC 30011; yeast culture isolate) and pathogenic (ATCC 30868; human corneal isolate) strains of *Acanthamoeba castellanii* Castellani were purchased from the American Type Culture Collection (ATCC). A keratitis sample isolated from an anonymized patient at the Department of Ophthalmology, Kyung Hee University Hospital (Seoul, Republic of Korea) was sent to our lab to confirm whether the keratitis sample was of Acanthamoebic origin. The clinical isolate shared 98% sequence homology with *Acanthamoeba* spp. based on the complete 18s rDNA sequence. All three *Acanthamoeba* strains were axenically cultured in PYG medium at 25°C and after 5 days, the culture media was collected. HCE cells were incubated at 37°C with 5% CO_2_ in endothelial cell growth media (KGM BulletKit; Lonza, Portsmouth, NH, USA). *P*. *aeruginosa* and *S*. *aureus* were cultured in Brain Heart Infusion media at 37°C, while *F*. *solani* was cultured in Sabouraud Dextrose (SD) media at 25°C. The culture media of *F*. *solani*, *P*. *aeruginosa*, and *S*. *aureus* were harvested after cultivating for 1 day.

### Cloning and peptide antibody production of chorismate mutase

Gene sequence of the *A*. *castellanii* Neff chorismate mutase (GenBank accession no: XM_004352878) was used to design the primers for chorismate mutase, which were subsequently used to amplify the coding sequence of *A*. *castellanii* Castellanii chorismate mutase by polymerase chain reaction. The primer information is as follows: 5’-ATGCGCTTCCTGCTCGCCTT-3’ (forward), and 5’-TCAATCGGCGTGCTGGACGG-3’ (reverse). The peptide sequence for the immunogen is as follows: NH_2_-C-RLQVETLNSEFNAGLPVPVQHAD-COOH. Synthesized peptide and the antibody raised against the peptide were purchased from AbFRONTIER (AbFRONTIER, Seoul, Korea). Briefly, two NZW rabbits were injected intradermally with 1.0 mg of peptide-KLH conjugates in complete Freund’s adjuvant on days 0, and 0.5 mg of peptide in incomplete Freund’s adjuvant on days 28, 42, and 56. The animals were bled on days 35 and 49. After the 3^rd^ immunization, antisera titer was assessed using an indirect ELISA with the peptide-BSA conjugates as coating antigens until the titer plateaued. After the last immunization, blood samples were acquired by cardiac puncture from each rabbit.

### *Acanthamoeba* specificity of the chorismate mutase antibody by western blot

*Acanthamoeba*-specificity of the chorismate mutase was confirmed using western blot as previously described [21]. Protein samples from the whole cell lysates and the culture media of *Acanthamoeba* spp., HCE cells, *F*. *solani*, *P*. *aeruginosa*, and *S*. *aureus* were prepared using a lysis buffer. After determining the concentration of each protein sample, 20 μg of cell lysates and 5 μg of conditioned media were resolved by SDS-PAGE and transferred onto a nitrocellulose membrane. After blocking the membrane with 5% skim milk in TBST for 1 h at RT, membrane was incubated overnight at 4°C with the chorismate mutase polyclonal antibody (1:1000). Membrane was washed with TBST, then incubated with horseradish peroxidase (HRP)-conjugated anti-rabbit antibody (1:5000) (Sigma-Aldrich, St. Louis, MO, USA) at RT for 1 h. Bands were developed using enhanced chemiluminescence (ECL; Thermo Fisher, MA, USA) on an x-ray film in the darkroom.

### Immunocytochemistry

HCE cells were seeded at a density of 3 × 10^5^ cells/well on a six-well plate and incubated in KGM BulletKit media at 37°C for 24 h. After incubation, *A*. *castellanii* (ATCC 30868) trophozoites were added to HCE cells at a density of 5 × 10^5^ cells/well, and incubated at 37°C for 4 h. After incubation, HCE cells and *A*. *castellanii* were fixed with 100% methanol for 5 min at RT and permeabilized using 0.2% Triton X-100 in phosphate-buffered saline (PBST) for 10 min at RT. After washing with PBS, cells were blocked using 1% bovine serum albumin in PBS for 30 min at RT. The cells were incubated overnight at 4°C with chorismate mutase antibody (1:200), washed with PBST, and subsequently incubated with mouse-anti-rabbit IgG-CFL-488 antibody (1:400) (Sigma-Aldrich, St. Louis, MO, USA) for 1 h at RT. Cells were washed with 1X PBS and stained with VECTASHIELD^®^ Mounting Medium with DAPI (Abcam, Burlingame, CA, USA). Images were acquired using fluorescent microscopy (Leica DMi8, Wetzlar, Germany).

### Enzyme-linked immunosorbent assay (ELISA)

IgG antibody levels specific to *A*. *castellanii* were determined by ELISA. A checkerboard titration of each antigen and serum were designed for the optimal antigen concentration (100 to 0.0001 μg/μl) and serum dilutions (1:5 to 1:5000). Cell lysates and the conditioned media of *Acanthamoeba* were used as coating antigens. Briefly, in 96-well microplates, each well was coated with the antigen dissolved in carbonate coating buffer (0.1 M sodium carbonate, pH 9.5) at 4°C overnight. Plates were washed three times with PBS containing 0.05% Tween 20 (PBST) and blocked with 0.2% gelatin for 1 h at 37°C. All antibodies were diluted in PBS. Diluted samples were incubated at 37°C for 1 h and plates were washed. HRP-conjugated goat anti-rabbit IgG (Cusabio Co. Ltd, Wuhan, China) was added at 1:1000 dilution and incubated for 1 h at 37°C. O-phenylenediamine (OPD; Zymed, San Francisco, CA) was dissolved in citrate-phosphate buffer (pH 5.0) with 0.03% H_2_O_2_ and subsequently used as substrate. The optical density values at 450 nm were read using an EZ Read 400 microplate reader (Biochrom Ltd., Cambridge, UK). Sera of naïve rabbits were used as negative control.

## Results

### Identification of chorismate mutase in *A*. *castellanii*

**Fig 1:**
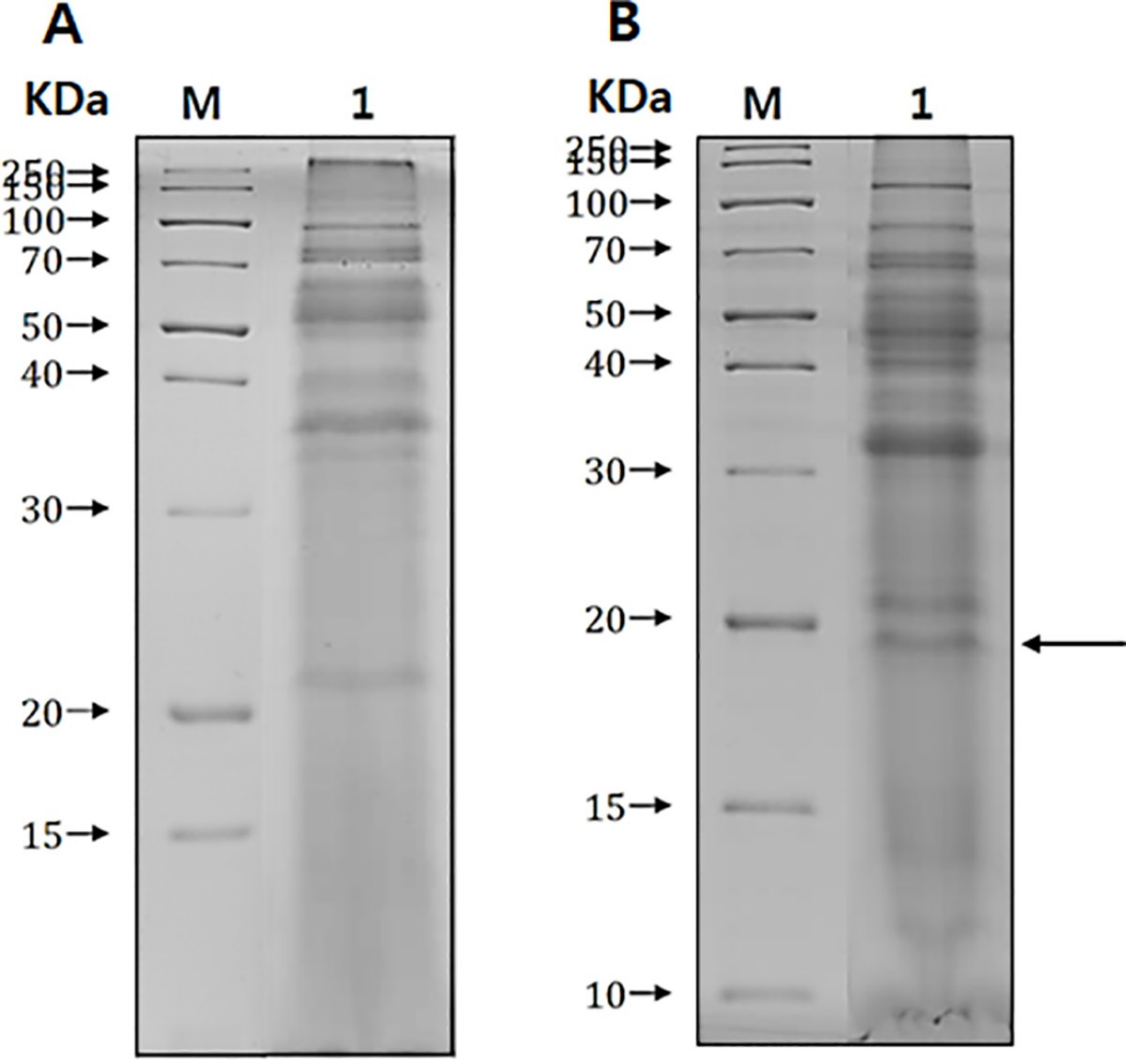
Secretory proteins of *A*. *castellanii*. Secretory proteins between non-pathogenic *Acanthamoeba* (ATCC 30011) (A) and pathogenic *Acanthamoeba* (ATCC 30868) (B) were compared by SDS-PAGE. M; protein size marker, lane 1; conditioned media of *Acanthamoeba*. An arrow indicates a specific protein band of pathogenic *Acanthamoeba* origin.

**Fig 2:**
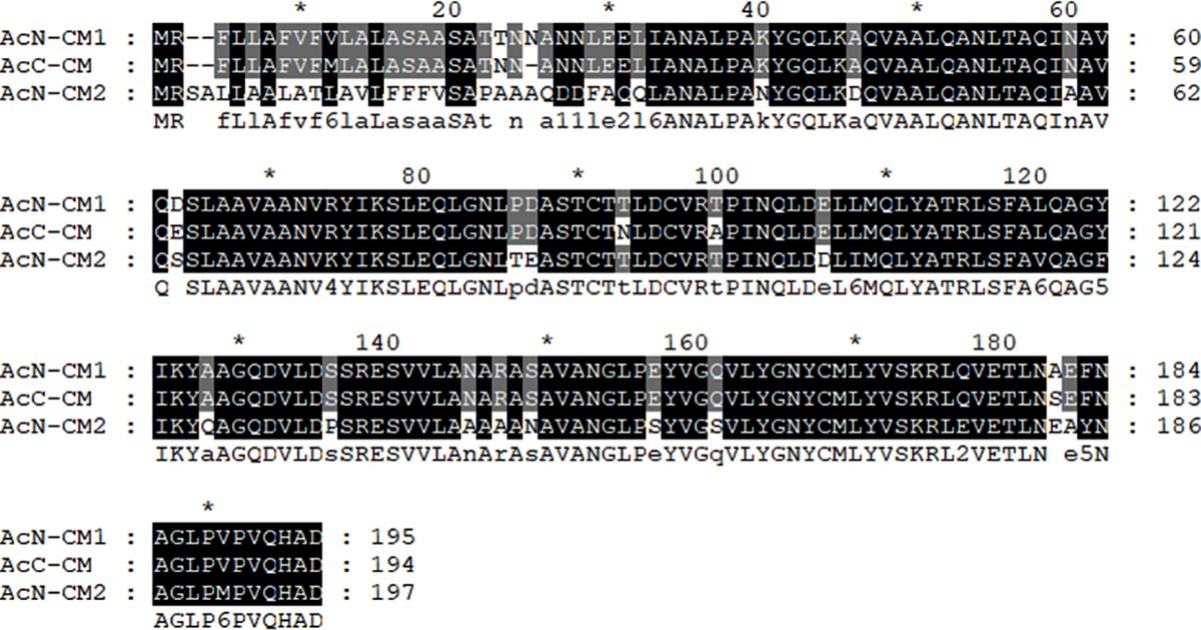
Alignment of the chorismate mutase amino acid sequences of *Acanthamoeba* spp.. Aligned amino acid sequences of *A*. *castellanii* Neff chorismate mutase 1 (ELR23397), *A*. *castellanii* Neff chorismate mutase 2 (ELR15898), and *A*. *castellanii* Castellani chorismate mutase (MN630520) were compared. ClustalX multiple sequence alignment was used to produce the alignment, and the degree of conservation is represented by different shading. AcN; *Acanthamoeba castellanii* Neff, AcC; *Acanthamoeba castellanii* Castellani, CM; chorismate mutase.

Secretory proteins isolated from both non-pathogenic (Fig 1A) and pathogenic (Fig 1B) *A*. *castellanii* were resolved by SDS-PAGE and compared for analysis. As illustrated in Fig 1, several protein bands that were specifically present only in the pathogenic *Acanthamoeba* strain were observed. One such protein, as indicated by the arrow in Fig 1B, was selected for identification. Mass spectrometry and NCBI database search results revealed that the selected protein showed the highest similarity with the chorismate mutase of *A*. *castellanii* Neff. In this study, the full-length open reading frame of chorismate mutase from pathogenic *A*. *castellanii* Castellani was identified by polymerase chain reaction (GenBank accession number MN630520). The gene consists of 585 bp and encodes 194 amino acids with a calculated mass 21.34 kDa. Protein homology search results revealed that this protein showed 96.9% similarity with chorismate mutase 1 (putative) of *A*. *castellanii* Neff and 71.6% similarity with chorismate mutase 2 (subfamily protein) of *A*. *castellanii* Neff (Fig 2 and Table 1). Peptide analysis was performed using the SignalP 4.1 server database, which predicted the presence of the signal peptide at amino acid positions 1 to 19.

**Table 1:** Identities of chorismate mutase amino acids as determined by protein BLAST.

Name	Accession No.	Length (aa)	Identities
AcC-CM	MN630520	194	194 (100%)
AcN-CM1	ELR23397	195	189 (96.9%)
AcN-CM2	ELR15898	197	144 (71.6%)
Fo-CM	EWY95915	223	34 (13.7%)
Fo-CM2	EWY95914	266	37 (13.3%)
Pa-CM	OHQ59631	185	36 (16.0%)
Pa-CM2	KJJ16793	365	37 (9.3%)
Sa-CM	OHS92057	363	30 (6.7%)

AcC; *Acanthamoeba castellanii* Castellani, CM; chorismate mutase, AcN; *Acanthamoeba castellanii* Neff, Fo; *Fusarium oxysporum*, Pa; *Pseudomonas aeruginosa*, Sa; *Staphylococcus aureus*.

### Production of chorismate mutase antibody

**Fig 3:**
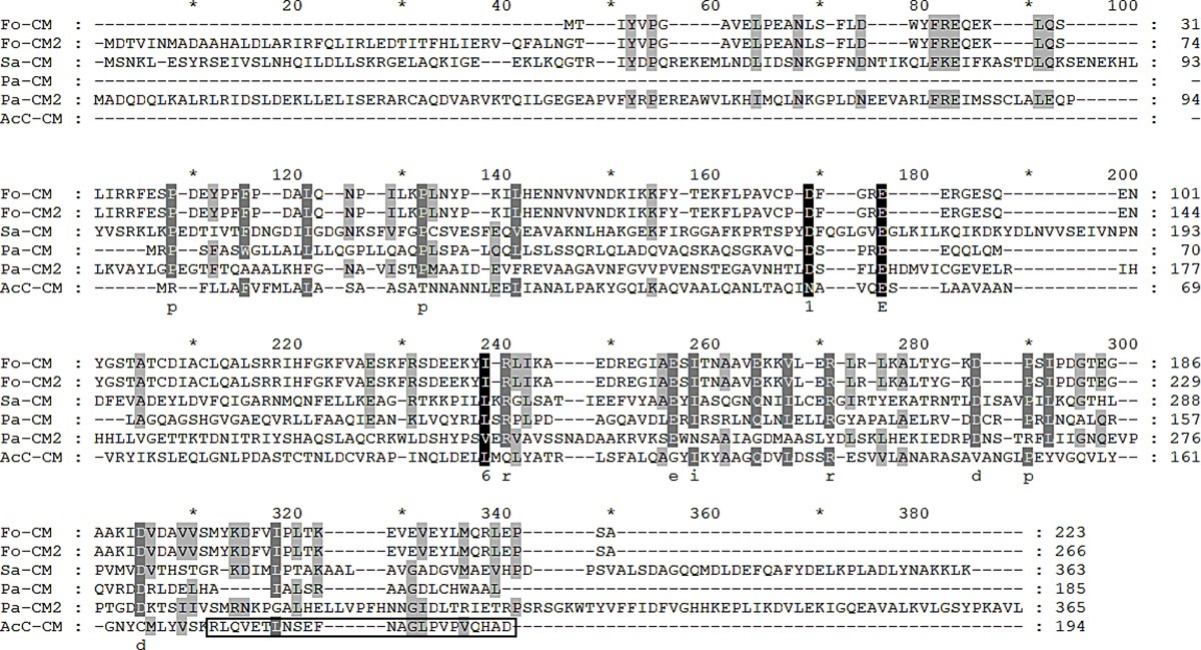
Alignment of the chorismate mutase amino acid sequences from different organisms. Aligned amino acid sequences of *F*. *oxysprum* chorismate mutase (EWY95915), *F*. *oxysprum* chorismate mutase 2 (EWY95914), *S*. *aureus* chorismate mutase (OHS92057), *P*. *aeruginosa* chorismate mutase (OHQ59631), *P*. *aeruginosa* chorismate mutase 2 (KJJ16793), and *A*. *castellanii* Castellani chorismate mutase (MN630520) were compared. The boxed sequence corresponds to the antigen peptides used to raise the anti-chorismate mutase polyclonal antibody. Fo; *Fusarium oxysporum*, Sa; *Staphylococcus aureus*, Pa; *Pseudomonas aeruginosa*.

To assess the applicability of the chorismate mutase protein for AK diagnosis, we produced a polyclonal peptide antibody against the chorismate mutase of *A*. *castellanii*. Based on the hydrophobicity and antigenicity analysis profiles, two highly immunogenic epitopes were identified at amino acid position 127–134 and 172–194. Amino acid sequences of chorismate mutase from *Acanthamoeba* was compared with that of other causes of keratitis such as *Fusarium* spp., *Pseudomonas aeruginosa*, and *Staphylococcus aureus*. Protein homology search results revealed that chorismate mutase of *Acanthamoeba* showed 13.7%, 13.3%, 16.0%, 9.3%, and 6.7% similarity with chorismate mutase 1 or 2 of *F*. *oxysporum*, *P*. *aeruginosa*, and *S*. *aureus*, each respectively (Fig 3 and Table 1). Based on the amino acid homology search, amino acids at positions 172–194 were selected for peptide antibody production (boxed area in Fig 3).

### Confirming the specificity of a chorismate mutase peptide antibody

**Fig 4:**
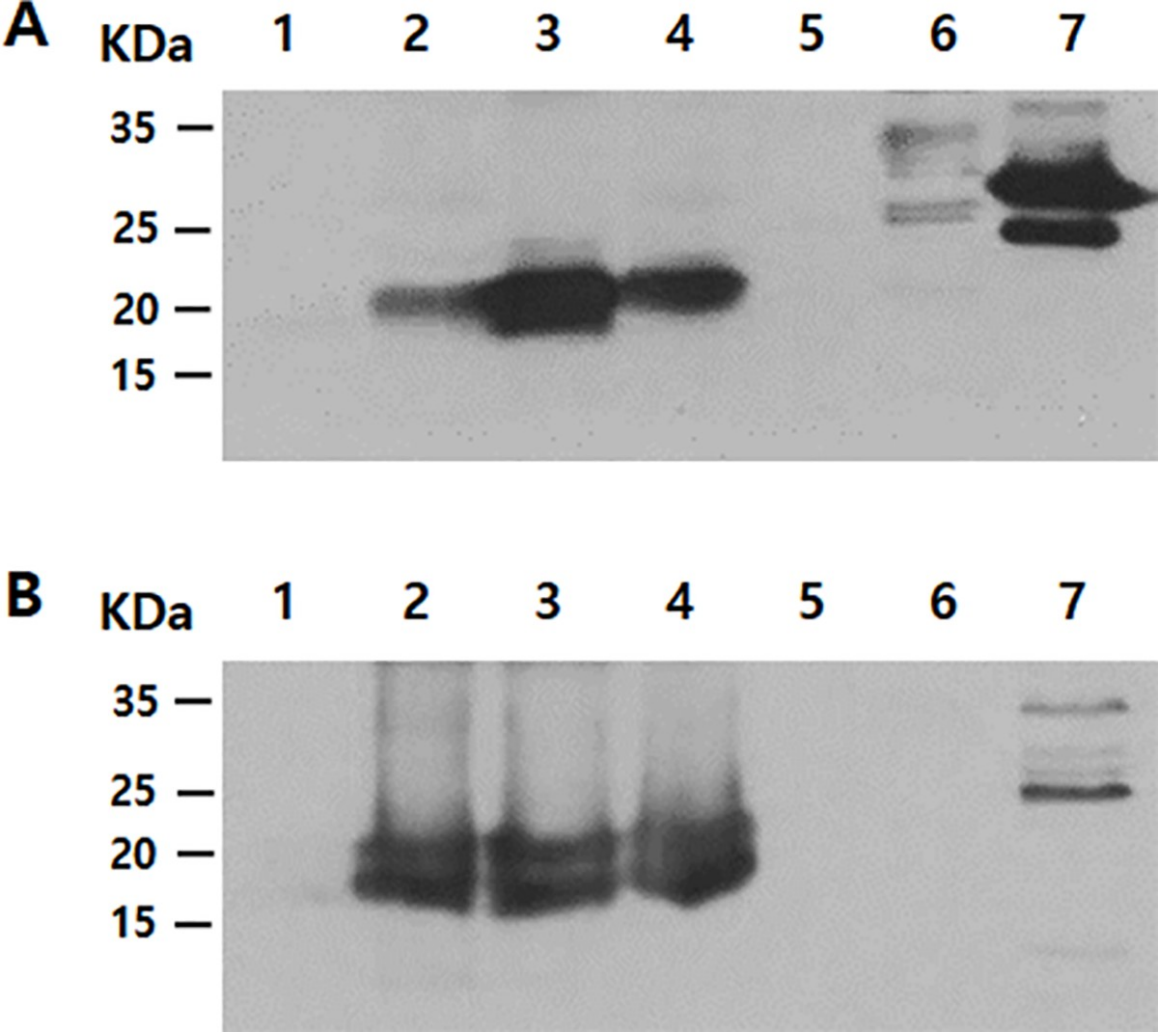
Specific antibody response of chorismate mutase. The differential diagnosis of *Acanthamoeba* keratitis was confirmed by western blot analysis of cell lysates (A) and conditioned media (B) using the polyclonal peptide antibody raised against chorismate mutase of *A*. *castellanii*. Lane 1: HCE cells, lane 2: non-pathogenic *Acanthamoeba*, lane 3: pathogenic *Acanthamoeba*, lane 4: clinical isolate of *Acanthamoeba*, lane 5: *F*. *solani*, lane 6: *P*. *aeruginosa*, and lane 7: *S*. *aureus*.

The specificity of the chorismate mutase antibody was demonstrated by western blot analysis using cell lysates and conditioned media of HCE cells, non-pathogenic *Acanthamoeba*, pathogenic *Acanthamoeba*, *Acanthamoeba* spp. isolated from a clinical sample, *F*. *solani*, *P*. *aeruginosa*, and *S*. *aureus* (Fig 4). Western blot results revealed that the chorismate mutase antibody did not react with HCE cell lysate and conditioned media (lane 1 of Fig 4A and 4B) but reacted with *Acanthamoeba* spp. cell lysate and conditioned media (lanes 2 to 4 of Fig 4A and 4B). The estimated sizes of the chorismate mutase protein and the secretory chorismate mutase in conditioned media were 21.34 kDa and 19.34 kDa, respectively. While both of these were detected from the conditioned media of *Acanthamoeba* spp., their presence was not confirmed in HCE cells, *F*. *solani*, *P*. *aeruginosa*, and *S*. *aureus* lysates (Fig 4B). Several non-specific bands were detected in the *P*. *aeruginosa* cell lysates, *S*. *aureus* cell lysate and its conditioned media (lane 7 of Fig 4A and 4B).

**Fig 5:**
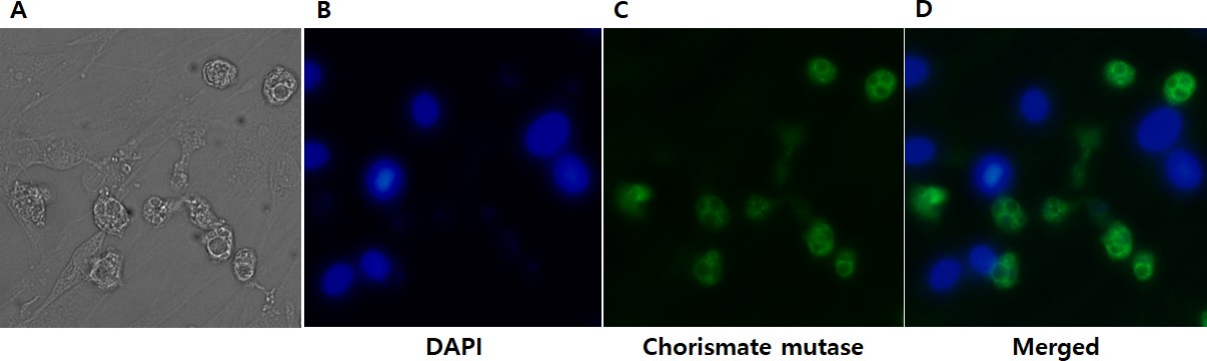
Immunocytochemistry using the *A*. *castellanii* chorismate mutase antibody. HCE cells cultured with *A*. *castellanii* trophozoites for 4 h were visualized under a fluorescent microscope. Bright-field (A), DAPI-stained (B), indirect immunocytochemical labeling (C), and merged images (D) were acquired at 400x magnification.

**Fig 6:**
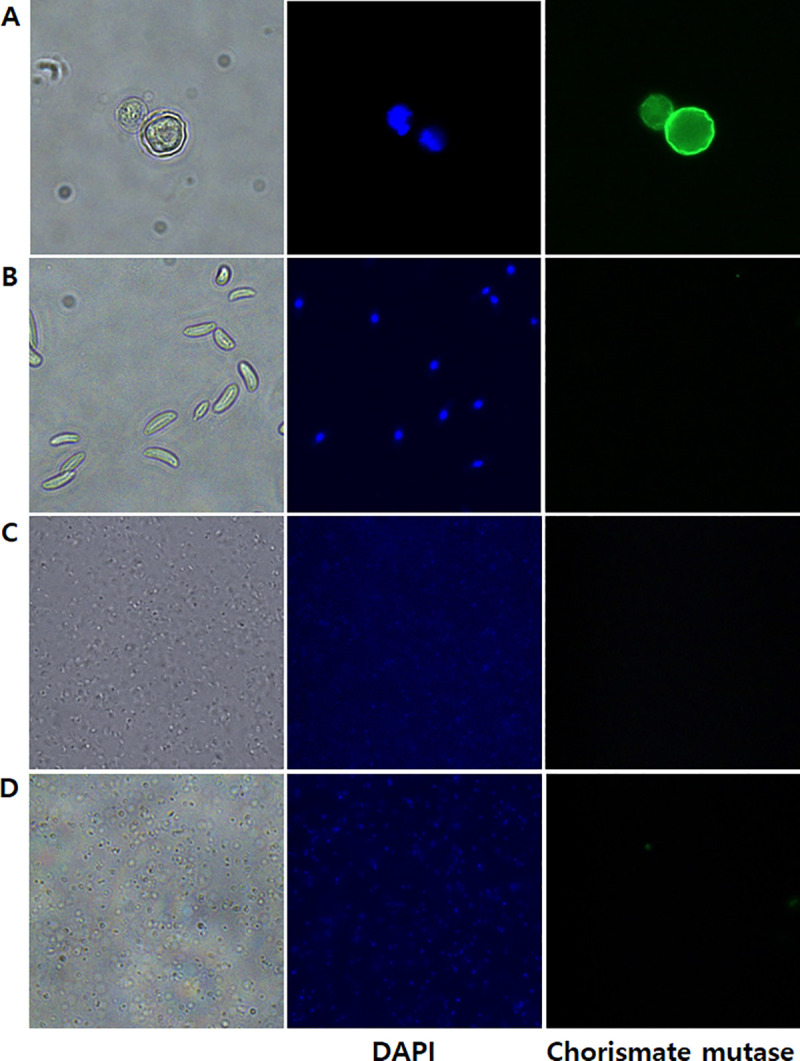
Confirming the species-specificity of the *A*. *castellanii* chorismate mutase antibody using immunocytochemistry. *A*. *castellanii* cysts (A), *F*. *solani* (B), *P*. *aeruginosa* (C), and *S*. *aureus* (D) were visualized under a fluorescence microscope. Bright-field, DAPI-stained, and indirect immunocytochemical labeling images were acquired at 400x magnification.

To further validate the chorismate mutase antibody specificity, immunocytochemistry was performed using HCE cells were cultured with *A*. *castelannii* (Fig 5). As expected, DAPI staining revealed the cellular nucleic acid contents of HCE cells (Fig 5B), whereas HCE cells remained unstained when incubated with chorismate mutase antibody (Fig 5C). On the contrary, strong interaction between the chorismate mutase antibody and *Acanthamoebae* trophozoites was observed (Fig 5C). Faint DAPI staining was observed from *Acanthamoeba*, which could be attributed to its smaller size in comparison to HCE cells. *A*. *castellanii* cysts were also detected with chorismate mutase antibody (Fig 6A), however, *F*. *solani*, *P*. *aeruginosa*, and *S*. *aureus* were not detected with that antibody (Fig 6B–6D). Overall, the chorismate mutase antibody demonstrated its potential for selective and differential detection of *Acanthamoeba* via immunocytochemistry.

### ELISA titer of anti-chorismate mutase antibody

**Fig 7:**
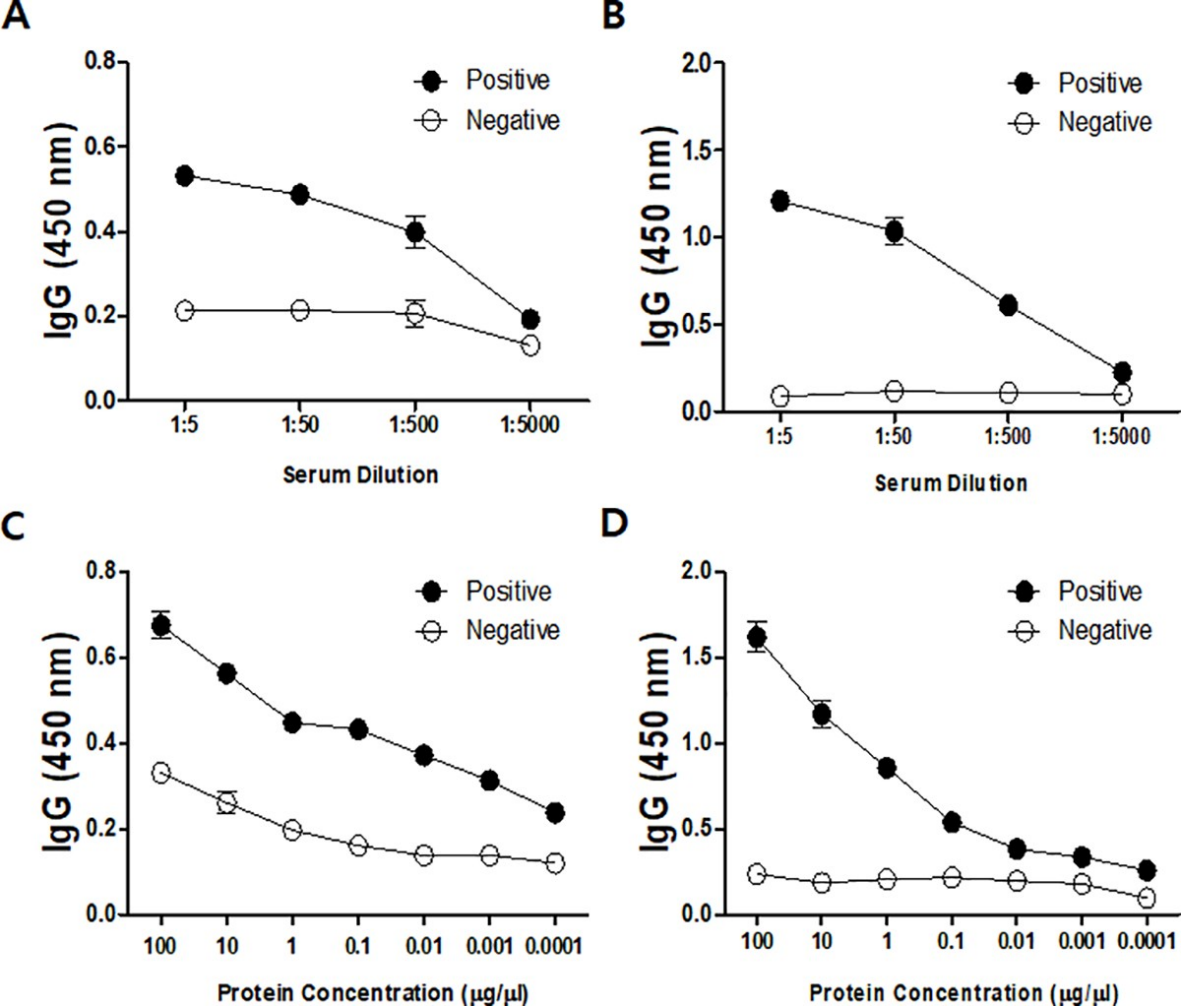
*A*. *castellanii* chorismate mutase-specific IgG response. The chorismate mutase polyclonal antibody was titrated using *A*. *castellanii* cell lysates (A) and conditioned media (B). Sensitivity of the antibody was also determined using cell lysates (C) and conditioned media (D). positive; immunized serum, negative; non-immuned serum.

As seen in Fig 7, serological analysis of immune sera indicated that a rabbit injected with *A*. *castellanii* chorismate mutase possessed *Acanthamoeba*-specific IgG. High IgG antibody titers were observed from both *A*. *castellanii* cell lysates (1/5000) and its conditioned media (1/5000) (Fig 7A and 7B). In addition, *Acanthamoeba*-specific IgG was demonstrated to be highly sensitive, with a detection limit as low as 0.1 ng for both *Acanthamoeba* cell lysate and the conditioned media (Fig 7C and 7D).

## Discussion

Specific detection and early diagnosis of *Acanthamoeba* infection are critical for an effective AK treatment. In recent years, culturing of live *Acanthamoeba* and amplifying its DNA by PCR have become the principal procedure for detecting *Acanthamoeba* and diagnosis of AK [12]. Nevertheless, pain infliction during the corneal sample acquisition process can be discomforting to the patients. Identifying a reliable method enabling rapid and accurate detection of *Acanthamoeba* has become an utmost priority, as delayed or inaccurate diagnosis consequentially leads to successful treatment failure with ensuing visual impairment.

In this study, we produced a chorismate mutase peptide antibody against *A*. *castellanii*, and confirmed the specific detection of *Acanthamoeba* among HCE cells and other causes of keratitis (Figs 4 and 5). This antibody could be suitable for detecting the presence of *Acanthamoeba* in human ocular infections. Chorismate mutase, though absent in mammals, is linked to the shikimate pathway, an essential process for the biosynthesis of aromatic compounds in bacteria, fungi, algae, and plants [22]. This enzyme is present in other microbial pathogens that contribute to AK such as *Fusarium* spp., *Pseudomonas* spp., and *Staphyloccus* spp. Amino acid sequence homology results have revealed that compared to the chorismate mutase of *A*. *castelanii*, the sequence homologies observed from *Fusarium*, *Pseudomonas*, and *Staphylococcus* were extremely low (Fig 3 and Table 1).

Previously, it was reported that *Acanthamoeba* must be present as trophozoites for binding to HCE cells since cysts are non-infective and do not facilitate cell binding [23–25]. Based on this rationale, immunocytochemistry analysis was conducted using only *A*. *castellanii* trophozoites and HCE cells (Fig 5). However, we confirmed that chorismate mutase antibody bound to *A*. *castellanii* cysts (Fig 6). Although our results demonstrated *Acanthamoeba*-specificity of the chorismate mutase antibody, confirming our results using other *Acanthamoeba* spp. that cause AK, and various clinical samples isolated from AK patients would further validate our findings.

Most of the time, corneal scrapes or corneal biopsy specimens are used to culture or identify *Acanthamoeba* for AK diagnosis [26]. In the future, corneal scrape sample acquisition for AK diagnosis could be replaced with other non-invasive methods such as corneal cell secretions or tears. *A*. *castellanii* has been found to secrete a large amount of chorismate mutase proteins (Figs 1 and 4B), which signifies the potential application of chorismate mutase antibody reported in this study for AK diagnosis. Two distinct bands were observed in the *Acanthamoeba* conditioned media. One possible explanation for this phenomenon is due to the signal peptide cleavage of the secretory protein, a 19 amino acids long peptide with a molecular weight of approximately 2 kDa. However, highly diluted protein samples by tears can limit the detection capabilities of the chorismate mutase antibody, which can lead to false-negative results. *A*. *castellanii* chorismate mutase-specific IgG revealed that the antibody was highly sensitive and could identify *A*. *castellanii* even at low concentrations (Fig 7C). For this study, we prepared 900 μg of protein cell lysate from 8 × 10^5^ trophozoites, implying that 0.1 ng of protein was prepared from a single trophozoite, and the lowest detection level of the antibody could be estimated as one trophozoite. Additional studies investigating the concentration of chorismate mutase in the patients’ tears and the minimum detectable concentration of the protein are needed before applying findings of the present study for AK diagnosis.

In conclusion, we produced an antibody against the secretory protein chorismate mutase of *A*. *castellanii* for the rapid and accurate identification of *Acanthamoeba*. Our *in vitro* studies suggest that this antibody is highly sensitive, indicating its potential application for differential AK diagnosis.

## Supporting information

S1 Raw image (PDF)
