# Supplementary material for: Chorismate mutase peptide antibody enables specific detection of Acanthamoeba
Source: PLoS One. 2021 Apr 23;16(4):e0250342. doi: 10.1371/journal.pone.0250342 (PMC8064552; doi:10.1371/journal.pone.0250342)
Supplement: S1 Raw image — (PDF) [file pone.0250342.s001.pdf]

Figure 4A

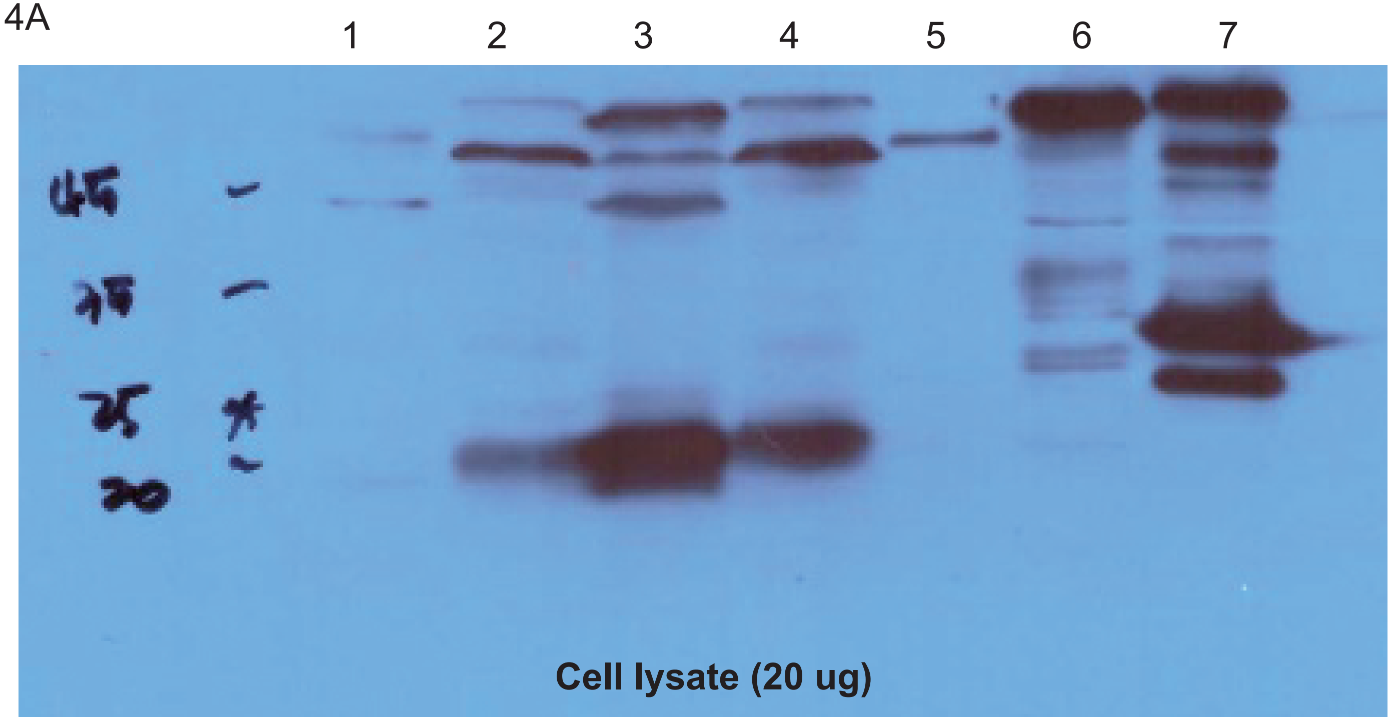

Figure 4B

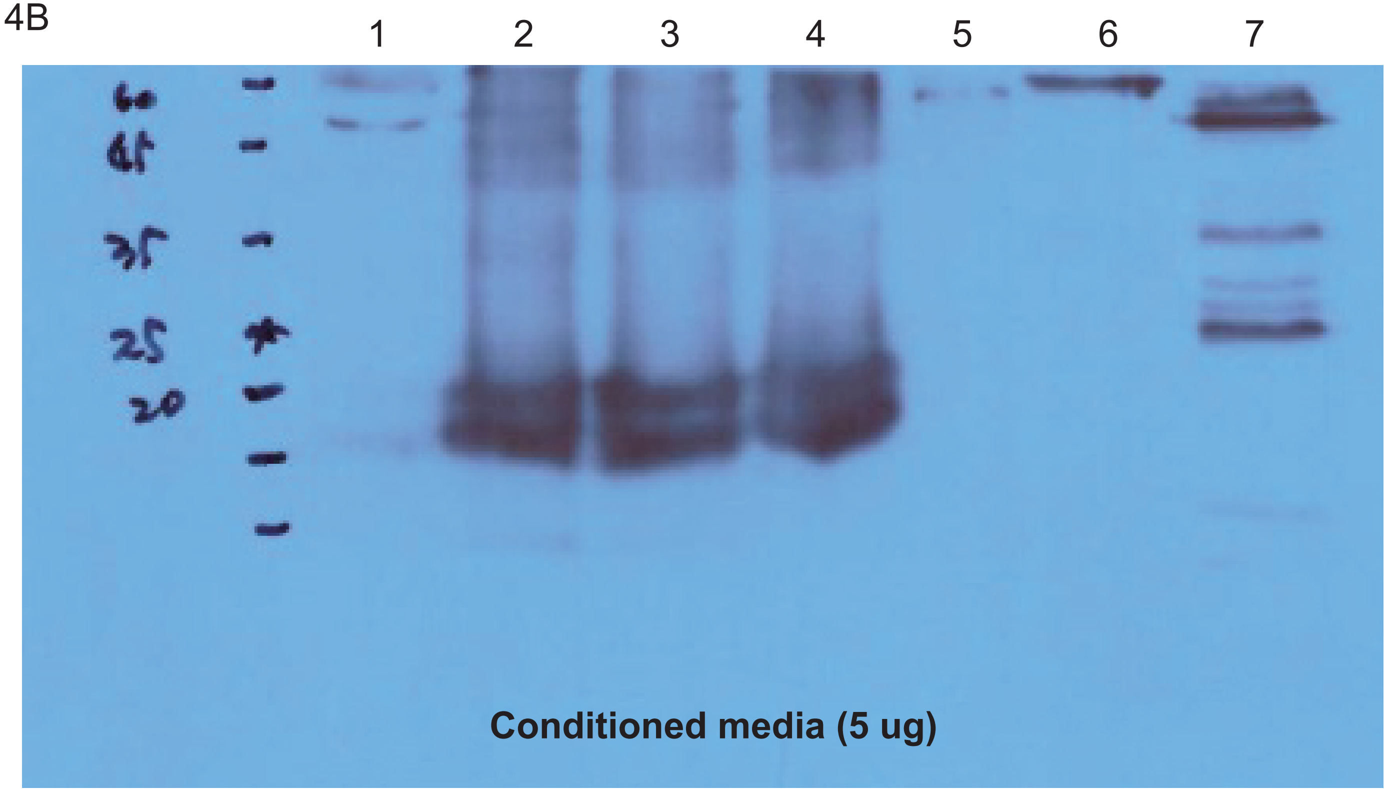

1. Human corneal epithelial cells
2. *Acanthamoeba castellanii* (#30011-nonpathogenic)
3. *Acanthamoeba castellanii* (#30868-pathogenic)
4. Clinical isolate (*Acanthamoeba* spp.)
5. *Fusarium solani*
6. *Pseudomonas aeruginosa*
7. *Staphylococcus aureus*
